# RPL24: a potential therapeutic target whose depletion or acetylation inhibits polysome assembly and cancer cell growth

**DOI:** 10.18632/oncotarget.2099

**Published:** 2014-06-12

**Authors:** Kathleen A. Wilson-Edell, Amanuel Kehasse, Gary K. Scott, Christina Yau, Daniel E. Rothschild, Birgit Schilling, Bianca S. Gabriel, Mariya A. Yevtushenko, Ingrid M. Hanson, Jason M. Held, Bradford W. Gibson, Christopher C. Benz

**Affiliations:** ^1^ Buck Institute for Research on Aging; Novato, CA, USA; ^2^ Master of Science in Biology Program; Dominican University; San Rafael, CA, USA; ^3^ Department of Pharmaceutical Chemistry, University of California, San Francisco, CA USA; ^4^ Oncology-Hematology Division, Department of Medicine, University of California, San Francisco, CA USA

**Keywords:** RPL24, eIF6, ribosome assembly, translation, HDACs, breast cancer

## Abstract

Partial loss of large ribosomal subunit protein 24 (RPL24) function is known to protect mice against Akt or Myc-driven cancers, in part via translational inhibition of a subset of cap(eIF4E)-dependently translated mRNAs. The role of RPL24 in human malignancies is unknown. By analyzing a public dataset of matched human breast cancers and normal mammary tissue, we found that breast cancers express significantly more RPL24 than matched normal breast samples. Depletion of RPL24 in breast cancer cells by >70% reduced cell viability by 80% and decreased protein expression of the eIF4E-dependently translated proteins cyclin D1 (75%), survivin (46%) and NBS1 (30%) without altering GAPDH or beta-tubulin levels. RPL24 knockdown also reduced 80S subunit levels relative to 40S and 60S levels. These effects on expression of eIF4E-dependent proteins and ribosome assembly were mimicked by 2-24 h treatment with the pan-HDACi, trichostatin A (TSA), which induced acetylation of 15 different polysome-associated proteins including RPL24. Furthermore, HDAC6-selective inhibition or HDAC6 knockdown induced ribosomal protein acetylation. Via mass spectrometry, we found that 60S-associated, but not, polysome-associated, RPL24 undergoes HDACi-induced acetylation on K27. Thus, RPL24 K27 acetylation may play a role in ribosome assembly. These findings point toward a novel acetylation-dependent polysome assembly mechanism regulating tumorigenesis.

## INTRODUCTION

Control of protein synthesis is commonly dysregulated in cancer, most frequently by mutational activation of the phosphoinositide 3-kinase, protein kinase B/Akt/mammalian target of rapamycin (PI3K/Akt/mTOR) pathway. The PI3K/Akt/mTOR pathway promotes cell survival and growth, by inducing the phosphorylation of the small (40S) ribosomal subunit protein S6 (RPS6) and the eukaryotic initiation factor 4e binding protein 1 (4eBP1). These events stimulate polysome (polyribosome) assembly and increase cap-dependent (eIF4E-dependent) translation of tumorigenic mRNAs [1-4].

Recent studies indicate that other pathways, in addition to the PI3K/Akt/mTOR pathway, can cause translational dysregulation in cancer. For example, the rRNA methyltransferase WBSCR22 is involved in the biogenesis of the 40S ribosomal subunit and is overexpressed in invasive breast cancer [5]. The large ribosomal subunit protein 24 (RPL24) is another translation factor previously linked to tumorigenesis. Homozygous RPL24 deficiency is lethal in mice. In contrast, RPL24 haploinsufficient mice are viable with specific eye, skeletal, and coat pigment defects [6]. Interestingly, these RPL24 haploinsufficient mice show greater survival from Akt-induced lymphomagenesis. This protection is associated with an overall decrease in thymocyte protein synthesis [7]. Likewise, RPL24 haploinsufficient mice are protected from Myc-driven tumorigenesis. This same study demonstrated that Myc-induced tumorigenesis arises by increased cap-dependent translation that is also prevented by RPL24 haploinsufficiency [8]. In studies of human lung adenocarcinoma cells depleted of RPL24 by RNA interference, and in RPL24 haploinsufficient mouse embryonic fibroblasts (MEFs), RPL24 reduction is associated with increased p53 expression [9], suggesting that the prevention of tumorigenesis by reduced RPL24 may also depend on a p53-dependent checkpoint mechanism.

A full understanding of the role of RPL24 in tumorigenesis requires mechanistic elucidation of how RPL24 interacts with other ribosomal proteins and translation factors. RPL24 is one of the later proteins to be incorporated into the large ribosomal subunit, where it then regulates the joining of the 60S subunit to the small 40S subunit [10-12]. Crystallography of the *Tetrahymena thermophilis* 60S ribosomal subunit and cryo-electron microscopy reconstruction of the *Saccharomyces cerevisiae* 60S indicate that RPL24 resides on a surface of the 60S ribosomal subunit close to where the eukaryotic initiation factor 6 (eIF6) contacts the 60S [13-15]. The anti-assembly factor, eIF6, binds to the pre-60S ribosomal subunit and prevents premature association of 60S with the 40S subunit. Following 60S maturation, eIF6 is released, allowing for the joining of the 40S sand 60S subunits to form the 80S ribosome and further assembly of polysomes [16-20].

Given the known role of RPL24 in murine tumorigenesis and therapeutic interest in eIF4E-driven human breast cancers [21], we asked if RPL24 expression is also altered during human breast tumorigenesis, and observed that most human breast cancers overexpress RPL24 relative to normal breast tissue. We then demonstrated that RPL24 depletion in breast cancer cells reduces their growth and viability in association with selectively impaired expression of cap-dependent proteins needed for survival and proliferation, while also inhibiting 80S ribosome and polysome assembly by preventing eIF6 release from the 60S subunit. We also showed that 2-24 h treatment with a pan-inhibitor of class I and II histone deacetylases, trichostatin-A (TSA), mimics the above effects of RPL24 depletion, inducing 60S subunit-associated acetylation of RPL24 at K27. TSA also induced acetylation of polysomal RPL24 at K93 and 14 other ribosomal proteins. Comparison of pan-, class-, and isotype-selective HDACi's suggested that HDAC6 controls total acetylation levels of ribosomal proteins, a conclusion supported by HDAC6 knockdown studies.

## RESULTS

### RPL24 expression is transcriptionally upregulated during human breast tumorigenesis

**Figure 1:**
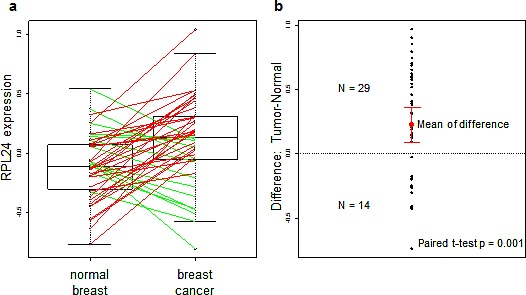
RPL24 expression is transcriptionally upregulated during human breast tumorigenesis RPL24 expression levels were analyzed from the dataset presented in [22]. (a) Box plot of RPL24 expression levels in patient-matched breast carcinoma and normal breast tissues. Lines connect paired data from each patient; and line color reflects relative levels of RPL24 in each paired sample (red: tumor > normal; green: normal > tumor). (b) Differences in RPL24 expression levels between each breast carcinoma and normal breast sample pair. The mean of the differences + SD are shown in red. P-value was obtained using a paired t-test.

Since RPL24 haploinsufficiency impairs the formation of both Akt-driven and Myc-driven murine malignancies [7-9], we looked for evidence that RPL24 upregulation may contribute to human tumorigenesis as well. To that end, we compared microarrayed samples of human cancers paired with their normal organ tissue samples. Using a public dataset of RNA profiles reported from 43 pairs of breast cancer and normal breast samples [32], we determined that approximately two-thirds of the breast cancers showed increased RPL24 transcript levels relative to their matched normal breast sample (Figure 1a). The entire group of tumor samples exhibited a significant 20% mean overall increase in RPL24 expression levels (p = 0.001), indicating that transcriptional upregulation of RPL24 commonly occurs in human breast tumorigenesis (Figure 1b).

### RPL24 knockdown reduces breast cancer cell viability while inhibiting cap (eIF4eE)-dependent expression of proliferation, survival and genome stability proteins

**Figure 2:**
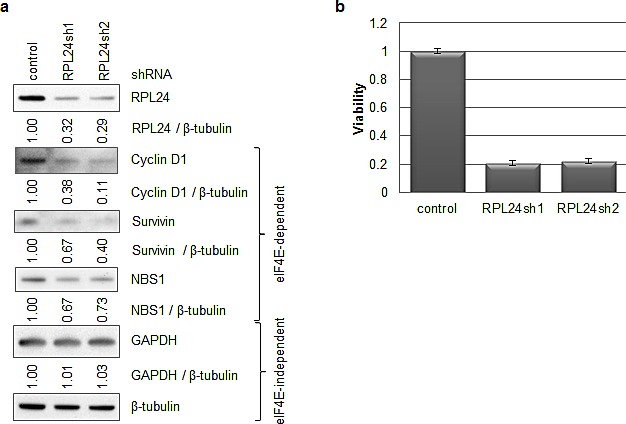
RPL24 knockdown reduces breast cancer cell viability while inhibiting cap (eIF4E)-dependent expression of proliferation, survival and genome stability proteins SKBR3 cells were infected with lentiviruses expressing a GFP control or RPL24-targeting shRNA. After one week of puromycin selection, cells were plated in 96-well plates for viability assays and lysates were taken in parallel for western blots. (a) Western blots were performed on lysates from an equal number of cells using antibodies toward the indicated proteins. Ratios of protein expression normalized to beta-tubulin levels were obtained using ImageJ software. (b) Viability assay readings were taken three hours after plating (day 0) and four days after plating (day 4). The day 4 results were normalized for plating efficiency using the day 0 values. Error bars represent three replicate samples.

Studies of RPL24 haploinsufficient mice protected from Myc-driven tumorigenesis revealed that dysregulated cap-dependent protein synthesis not only induces tumor formation but also results in cell cycle dysregulation and genomic instability [8]. Since the translation-dependent checkpoint mechanism remains undefined, we evaluated the impact of RPL24 depletion in a model human breast cancer cell line, SKBR3, sensitive to eIF4E-regulated and cap-dependent translation inhibition [33]. Two different RPL24-directed shRNA-expressing lentiviruses were used to decrease RPL24 protein expression by approximately 70% (Figure 2a). This resulted in a 5-fold (80%) reduction in SKBR3 cell viability measured after 4 days in culture (Figure 2b). Associated with RPL24 depletion and growth inhibition was a marked reduction in the expression of three different eIF4E-regulated and cap-dependent transcripts necessary for cell proliferation (75% reduction in cyclin D1), survival (46% reduction in survivin), and DNA repair and integrity (30% reduction in NBS1). Protein levels of two housekeeping genes not regulated by eIF4E [21, 33-35], GAPDH and β-tubulin, were not affected by RPL24 depletion (Figure 2a).

### RPL24 knockdown reduces 80S and polysome assembly while increasing 60S retention of eIF6

**Figure 3:**
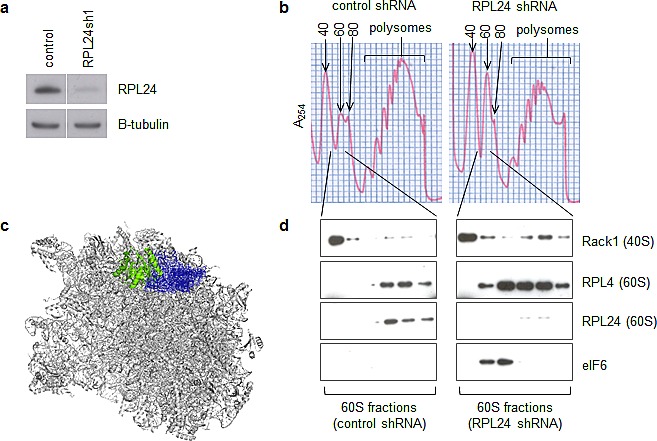
RPL24 knockdown reduces 80S and polysome assembly while increasing 60S retention of eIF6 (a,b,d) SKBR3 cells were infected with lentiviruses expressing a GFP control or RPL24-targeting shRNA for three days. (a) Western blots using the indicated antibodies were performed on total cell lysates to assess knockdown efficiency. (b) Lysates were applied to a continuous sucrose gradient (10-50%) and ultracentrifugation followed by fractionation was performed to separate ribosomal subunits and polysomes. (c) Pymol software was used to visualize the location of RPL24 (blue) relative to eIF6 (green) on the previously published structure of the 60S subunit in complex with eIF6 [13]. (d) Western blots using the indicated antibodies were performed on fractions from the 60S peaks using the indicated antibodies.

Since RPL24 depletion decreased the levels of three cap-dependently translated proteins, we next evaluated the impact of RPL24 knockdown on overall ribosome and polysome formation in these cells. We used polysome profiling, which utilizes continuous sucrose gradient fractionation to separate free 40S and 60S ribosomal subunits, 80S ribosomes, and polysomes (two or more ribosomes on one mRNA). The ratio of both 80S ribosomes and polysome peaks relative to free 40S and 60S ribosomal subunits was significantly reduced in SKBR3 cells following efficient RPL24 knockdown (Figure 3a,b). This observed increase in 40S and 60S subunits relative to 80S ribosomes implies a defect in 40S-60S joining induced by the RPL24 knockdown. Since eIF6 bound to the pre-60S subunit prevents joining of the 40S and 60S subunits [16-20] and occurs adjacent to RPL24 on 60S [13] (Figure 3c), we performed immunoblotting on all 60S-containing polysome fractions to evaluate the impact of RPL24 knockdown on eIF6 retention. Probing fractions corresponding to the area of the polysome profile near the 60S peak for Rack1, an obligatory 40S component, and RPL4, an obligatory 60S component, confirmed that these fractions do, in fact, encompass the 60 subunit. As expected, RPL24 levels were drastically decreased in SKBR3 cells expressing RPL24 shRNA. Associated with RPL24 knockdown was a striking increase in 60S-associated eIF6 (Figure 3d). To rule out the possibility that the observed 60S retention of eIF6 might be a false-positive or non-specific artifact of lentiviral expressed RPL24 shRNA, we overexpressed a functionally deficient truncation mutant of RPL24 that eliminates the last 17 amino acids. Polysome profiling of 293T cells expressing intact versus truncated RPL24 protein confirmed that truncated RPL24 can induce 60S retention of eIF6 (Figure S1).

### Ribosomal protein acetylation is induced by pan-HDACi and HDAC6-selective inhibitors

**Figure 4:**
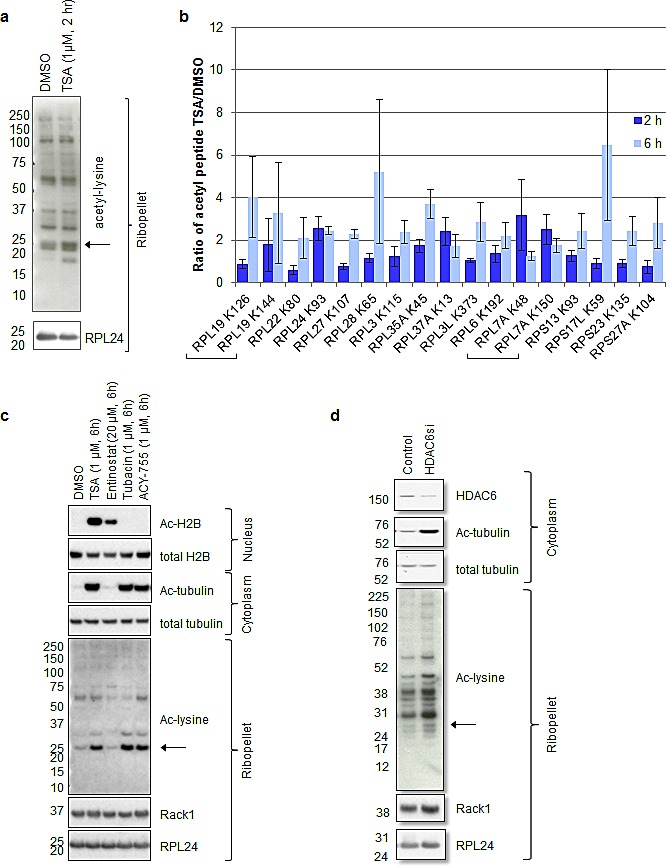
Ribosomal protein acetylation is induced by pan-HDACi and HDAC6-selective inhibitors (a-c) SKBR3 cells were treated with the indicated drugs for the indicated period of time. (d) SKBR3 cells were transfected with the indicated siRNAs and allowed to incubate for 72 hours. (a, c, d). The indicated western blots were performed in ribopellets, total cytoplasmic lysates, or nuclear extracts. (b) Mass spectrometry was performed on ribopellets as described in materials and methods and in Figure 6. The fold change in acetylated peptide to total peptide caused by TSA treatment is plotted. Only proteins that underwent at least a two-fold induction upon TSA treatment are shown. Error bars represent the standard error of the mean for three biological replicates.

Previous studies have shown that pan-inhibitors of class I and II histone deacetylases (pan-HDACi), like TSA, can rapidly destabilize a number of oncogenic transcripts including HER2 in a cycloheximide-dependent manner (Figure S2, [25, 36]). Since cycloheximide inhibits polysome formation, these results suggested that polysomes are possibly involved in HER2 mRNA decay. Thus, SKBR3 cells were treated with TSA to evaluate polysome protein acetylation and determine if, similar to RPL24 depletion, HDACi can affect ribosome assembly dynamics. To detect early (2 h) ribosome or polysome acetylation following HDACi treatment (1 μM TSA), SKBR3 polysomes were isolated using a discontinuous sucrose gradient as previously described [28]. Western blots using an antibody against acetylated lysine residues showed several TSA-induced bands, including a prominent TSA-induced acetyl-lysine protein band co-migrating with RPL24 (Figure 4a, indicated by arrow), while total RPL24 levels were not altered by TSA. Mass spectrometry studies indicate that 15 ribosomal proteins, 11 large subunit proteins (RPL24 included) and 4 small subunit proteins, underwent at least a two-fold induction in acetylation following 2h or 6h TSA treatment (1 μM) (Figure 4b, Figure S3a-q).

Like TSA, the HDAC6 (class IIb)-selective inhibitors, Tubacin and ACY-775, as well as HDAC6 siRNA, all induce tubulin acetylation as expected as well as ribosomal protein acetylation, including the band that co-migrates with RPL24 (Figure 4c,d, indicated by arrows). Although the class I-specific HDACi, Entinostat, induces histone H2B acetylation without acetylating tubulin, it does not alter ribosomal protein acetylation even at a dose of 20 μM (Figure 4c). Thus, the tubulin-acetylating effects of pan-HDACi, known to be mediated by inhibition or knockdown of HDAC6, correspond to the observed ribosome and RPL24 acetylation responses induced by TSA.

### Like RPL24 knockdown, HDACi reduces 80S assembly while increasing 60S retention of eIF6 and reduces expression of cap (eIF4E)-dependently translated proteins

**Figure 5:**
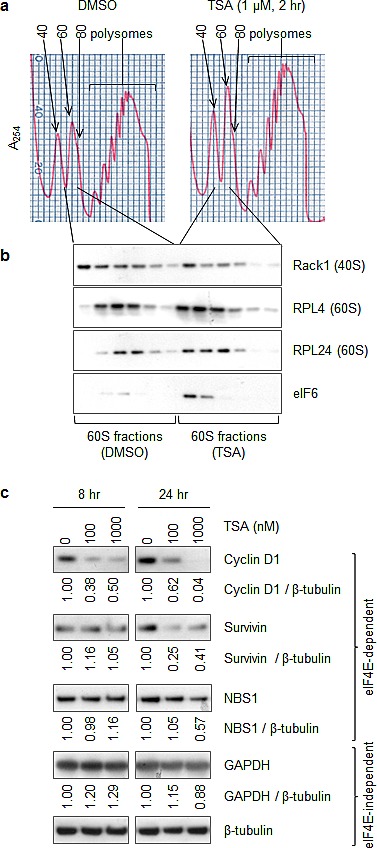
Like RPL24 knockdown, HDACi reduces 80S assembly while increasing 60S retention of eIF6 and reduces expression of cap (eIF4)-dependently translated proteins (a,b) SKBR3 cells were treated with TSA (1 μM, 2 h). (a) Polysome profiles were carried out as previously described. (b) Western blots using the indicated antibodies were performed in fractions representing the 60S subunits. (c) SKBR3 cells were treated with TSA for the indicated doses and times, and proteins were identified by western blotting as indicated. Ratios of protein expression normalized to beta-tubulin levels were obtained using ImageJ software.

Using continuous sucrose gradient fractionation of SKBR3 polysomes, 2 h culture treatment with TSA reduced 80S and polysome assembly (Figure 5a) while increasing 60S retention of eIF6 without reducing 60S RPL24 levels (Figure 5b). This result is comparable to that produced by RPL24 knockdown (Figure 3) or truncation (Figure S1). Furthermore, similar to RPL24 knockdown, 24 h TSA treatment reduced the expression of the cap(eIF4e)-dependently translated proteins cyclin D1, survivin, and NBS1 relative to the housekeeping proteins GAPDH and β-tubulin (Figure 5c). Shorter (8 h) TSA treatment reduced cyclin D1 levels but not survivin or NBS1 levels. The more rapid reduction of cyclin D1 levels was likely caused by the known effects of TSA on cyclin D1 transcription and transcript stability in addition to its effects on translation [37].

### HDACi enhances lysine (K27) acetylation on 60S RPL24

**Figure 6:**
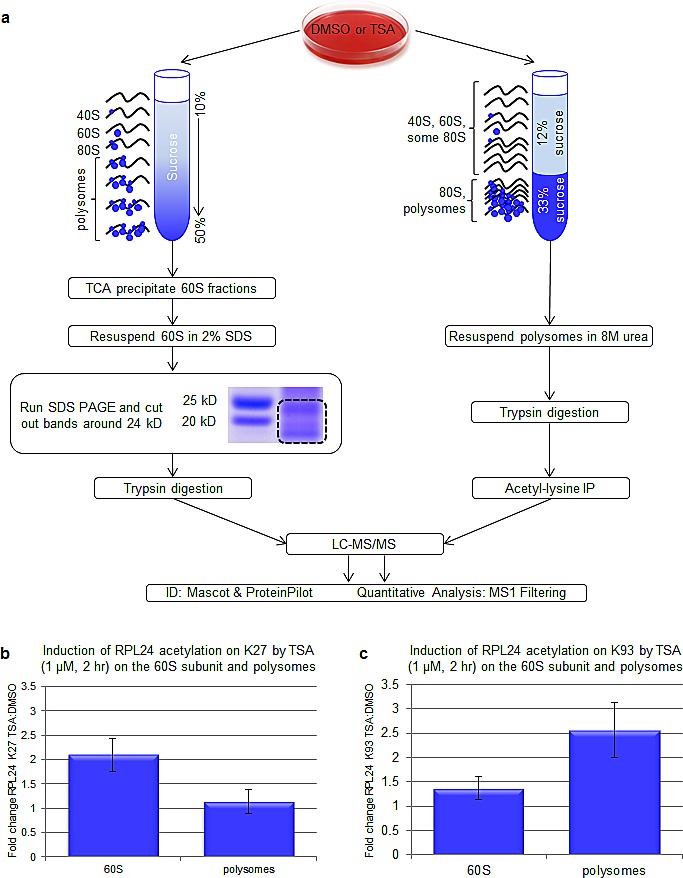
HDACi enhances lysine (K27) acetylation on 60S, but not polysomal, RPL24 (a) Schematic of mass-spectrometry-based techniques to analyze ribosomal protein acetylation. SKBR3 cells were treated with TSA (1 μM, 2 h or 6 h). To isolate 60S subunits, polysome profiles were performed and 60S fractions were TCA precipitated. Concentrated 60S samples were resolved on 4-12% bis-tris gels and RPL24-containing bands were excised and trypsin digested. In parallel, polysomes were isolated using a discontinuous sucrose gradient as described. Trypsin digestions and acetyl lysine immunoprecipitations were subsequently carried out. Mass spectrometry was performed on 60S-associated RPL24-containing gel bands or polysome-containing acetyl-lysine immunoprecipitations as described in the methods section. (b,c) On 60S-associated and polysome-associated RPL24, the fold induction caused by TSA (1 μM, 2 h) of K27 (b) or K93 (c)-acetylated peptide (normalized to total protein concentration) was plotted. Error bars represent the standard error of the mean for three biological replicates. Note: the data for K93 acetylation of RPL24 K93 is also shown in Figure 4b.

**Figure 7:**
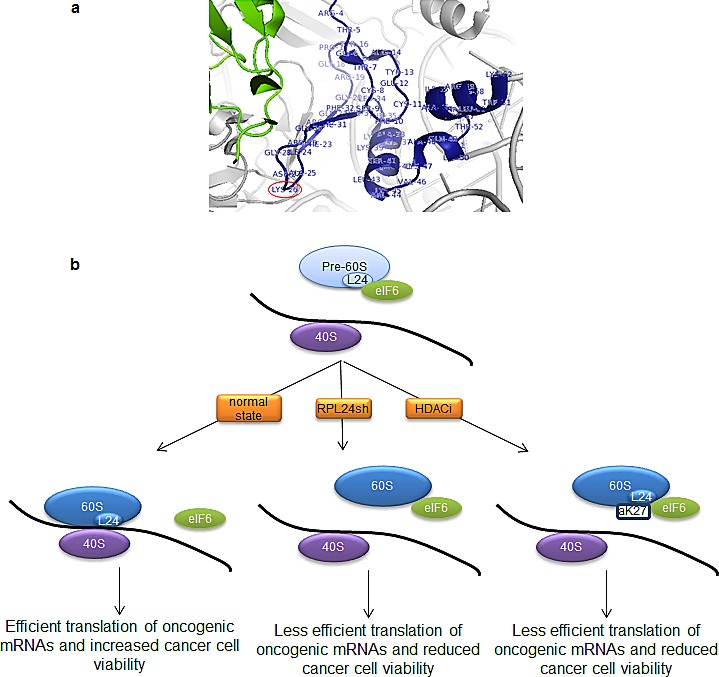
Schematic for modulation of ribosome assembly by RPL24 acetylation (a) A magnified portion of the RPL24 (blue)-eIF6 (green) interface, visualized with Pymol software, from previous x-ray crystallography data [13], is shown (zoomed out view shown in Figure 3c). T. thermophilia RPL24 residues are labelled and K26, which resides in a region of RPL24 homologous to where human K27 resides, is circled. (b) eIF6 binds to the pre-60S near RPL24 to prevent premature association of the 40S and 60S ribosomal subunits; eIF6 is then released from the mature 60S, allowing it to join with the 40S to form the 80S ribosome. Our model indicates that either RPL24 depletion or TSA (HDACi)-induced RPL24 acetylation on K27 prevents eIF6 release and 80S formation.

Mass spectrometry studies were performed to identify sites of lysine (K) acetylation within RPL24 induced by HDACi. Continuous and discontinuous sucrose gradient fractionations were performed to isolate 60S subunits and total polysomes respectively. Polyacrylamide gel electrophoresis was then performed on 60S and polysome samples and RPL24-containing bands were excised, trypsin digested, and subjected to mass spectrometry (LC-MS/MS) (Figure 6a). Among several detected acetylated RPL24 peptides, two were increased by TSA treatment; TDGKacVFQFLNAK (acetyl-K27) and AITGASLADIM*AKacR (acetyl-K93), where the internal lysines in both peptides are N-acetylated (Kac). As the MS experiments of the 60S polysome were performed after in-gel digestion the methionine residue of the second peptide was predominantly oxidized (M*=M+16), as commonly observed during SDS PAGE processing. In independent, in-solution digestion experiments, we also identified the corresponding non-oxidized form of acetylated peptide AITGASLADIMAKacR with correlating MS/MS fragmentation pattern. Representative spectra are shown for TDGKac VFQFLNAK (acetyl-K27) and AITGASLADIM*AKacR (acetyl-K93)) (Figure S4a,b). In 3 biological replicate experiments, the amount of K27-acetylated RPL24, normalized to total protein concentration within the 60S subunit, was increased at least 2-fold within 2 h of TSA treatment. However, there was no significant induction of RPL24 K27 acetylation found within polysomes (not containing 60S subunits) (Figure 6b). In contrast, RPL24 K93 acetylation within the 60S subunits was not significantly changed by TSA treatment, yet K93 acetylation was induced 2.5-fold within RPL24 associated with polysomes (Figure 6c). Given the proximity of the T. thermophilia RPL24 K26 site (that resides in a homologous region to human K27) to eIF6 (Figure 7a), these findings implicate involvement of the TSA induced acetylation of RPL24 at K27 in preventing 40S-60S subunit joining and 60S retention of eIF6.

## DISCUSSION

Consistent with the role of RPL24 in protecting against Akt-driven and Myc-driven murine tumorigenesis [7, 8], the present study provides new evidence that, not only is elevated RPL24 expression common in human breast tumors, but depletion or structural alteration of RPL24 can significantly impair human breast cancer cell viability (Figure 1,2). Our results indicate that this could occur through a reduction in cap(eIF4E)-dependent translation of proteins essential for cancer cell proliferation (e.g. cyclin D1), survival (e.g. survivin) and genome repair (e.g. NBS1) (Figure 2). Our findings also indicate that the mechanism of action by which RPL24 deficiency reduces expression of these proteins essential for cancer cell viability involves failure of 40S and 60S ribosomal subunit joining into functional 80S ribosomes by retention of eIF6 on 60S subunits (Figure 3).

Although RPL24 is an essential ribosomal protein, partial (up to 70%) depletion of RPL24 does not reduce the expression of all proteins but preferentially inhibits expression of eIF4E-regulated and cap-dependently translated proteins. In particular, the translation of housekeeping genes like GAPDH and β-tubulin remained unaffected when assessed by protein levels normalized to total cell number (Figure 2). In contrast, cyclin D1, survivin, and NBS1 are among several well-known growth, survival and tumorigenic transcripts with highly structured 5' untranslated regions (UTRs) whose cap-dependent ribosomal translation is tightly regulated by eIF4E; and their synthesis was reduced by as much as 75% following partial depletion of RPL24. These results raise the possibility that RPL24 depletion changes ribosome structure or recruitment factors in a manner that causes ribosomes to be recruited to cap-dependently translated mRNAs less efficiently.

As demonstrated in this study and schematically illustrated in Figure 7b, treatment of breast cancer cells with a pan-HDACi like TSA induces recruitment of eIF6 to 60S subunits along with acetylation of RPL24 at K27, likely impairing 40S-60S joining. These phenotypes mimic RPL24 knockdown (Figure 3, Figure 5). Consistent with this finding, the crystal structure of the *T. thermophilia* 60S subunit [13] reveals that RPL24 K26, which closely aligns with human RPL24 K27, is adjacent to eIF6 (Figure 3c, 7a). While we detected multiple sites of acetylation on RPL24, only K27 acetylation was induced by HDACi in association with the 60S subunit. Similar to RPL24 depletion, 24 h of HDACi treatment reduced the expression of cyclin D1, survivin, and NBS1 relative to GAPDH and β-tubulin (Figure 5c), a possible secondary consequence of the 60S acetylation and ribosome assembly failure noted as early as 2 h after TSA treatment. These timing differences in responses to the same TSA treatment suggest that although ribosome assembly is inhibited rapidly, it takes hours for the effects of such a functional impairment in ribosome assembly to manifest as a significant reduction in the pools of proteins synthesized by cap-dependent translation.

Our findings point toward the existence of an as yet unrecognized endogenous and acetylation-dependent mechanism that regulates both polysome assembly and cap-dependent protein translation. Including RPL24, we identified fifteen ribosomal proteins that undergo acetylation induction two-fold or more as a rapid consequence of HDACi treatment. These proteins are also potential contributors to this acetylation-dependent control of ribosome assembly. Like pan-HDACi treatment, enzymatic inhibition or knockdown of cytoplasmic HDAC6 appears to be sufficient to induce polysomal protein acetylation, in contrast to a class I-selective HDACi like Entinostat which has no such affect (Figure 4). Since only 2-6 h treatment with an HDAC6-selective inhibitor induces near maximum levels of ribosome protein acetylation, this posttranslational structural change is likely due to a direct cytoplasmic HDAC effect on ribosomal proteins rather than any indirect effect mediated by HDAC regulated nuclear gene expression.

In view of the current lack of any drug-like agent that can specifically target RPL24, our study also points toward RPL24 as a previously unrecognized subcellular target potentially contributing to the known anticancer activity of two approved cancer therapeutics, vorinostat and romidepsin, that are HDACi's like TSA, not to mention contributing to the anticancer mechanisms of investigational HDAC6-selective inhibitors like ACY-775. The fact that homozygous loss of RPL24 is embryonic lethal [6] might cause concern about developing a target-specific anti-RPL24 cancer therapeutic. However, it is important to recall that the homozygous deletion of many well-established breast cancer drug targets, such as human epidermal growth factor receptor (HER2), mTOR, and the catalytic domain of PI3K, are also embryonic lethal [38-41], yet this fact did not preclude or even delay the successful clinical development of therapeutics selective for these targets. The fact that RPL24 haploinsufficiency is compatible with normal murine lifespan is additionally reassuring from the standpoint of developing an anti-RPL24 therapeutic, and points to the established fact that all approved anticancer agents rely on *in vivo* definition of an optimal therapeutic window, whereby anticancer efficacy vastly exceeds host toxicity. Given that RPL24 haploinsufficiency protects mice from Akt- and Myc-driven tumorigenesis [7, 8], it is interesting to note that at least 40% of basal-like breast cancers overexpress Akt or Myc [42], indicating that anti-RPL24 therapeutics could be effective against basal-like and other clinically aggressive subsets of human breast cancer.

## METHODS

### Analysis of RPL24 expression in patient-matched breast carcinoma and normal breast tissue

Expression data from 43 patient-matched breast carcinoma and normal breast tissue samples assayed on Affymetrix U133A microarrays (GSE15852) was obtained from the Gene Expression Omnibus (GEO) [22]. Raw data was RMA-normalized, annotated using its associated platform annotation file (GPL96-39578) and mean-centered. Expression levels of the RPL24 probe within the patient-matched tumor and normal samples were obtained and compared. Significance was assessed using the paired t-test.

### Cell culture

SKBR3 human breast cancer cells (American Type Culture Collection (ATCC), Rockville, MD) were grown in McCoy's 5A media supplemented with 10% fetal bovine serum (FBS) and L-glutamate. 293T cells (American Type Culture Collection (ATCC), Rockville, MD) were grown in DMEM with 10% FBS and L-glutamate.

### shRNA and retroviral infection

Lentiviral vectors containing shRNAs toward RPL24, TRCN0000117642/RPL24sh1/target sequence CCTGAAGTTAGAAAGGCTCAA and TRCN0000117643/RPL24sh2/target sequence GTGCATCTCTTGCTGATATAA, and a green fluorescent protein control RHS4459/control/target sequence TACAACAGCCACAACGTCTAT were purchased from Thermo Scientific (formerly Open Biosystems, Cincinnati, OH). shRNA expressing lentiviruses were produced as previously described [23-25]. Briefly, 293T cells were transfected with lentiviral vectors along with packaging vectors. One day later, media was changed to Optimem (Life Technologies, Grand Island, NY) and the virus was collected for two days and concentrated it as outlined previously [26, 27]. SKBR3 cells were infected in the presence of 6 μg/ml polybrene with a multiplicity of infection of ~2. One day after infection media was changed to regular growth media in the case of transient infections or growth media with 0.5 μg/ml puromycin in the case of stable transfections.

### siRNA transfection

The following siRNAs were purchased from (Thermo Scientific-Dharmacon, Chicago, IL): HDAC6-targeting smart pool (L-003499-00) and non-targeting control pool (D-001810-10-05). Lipofectamine 2000 (Life Technologies) was used to transfect SKBR3 cells per manufacturer's protocol. Cells were analyzed 72 hours after transfection.

### Viability assay

Cells infected with different shRNA-expressing lentiviruses were plated in 96-well plates at a density of 5,000 cells per well. Three hours after plating (T_0_), a base line viability reading was taken using the CellTiter-Glo Luminescent Cell Viability Assay (Promega, Madison, WI). Four days later (T_4_) another reading was taken using the same assay. For each treatment, each of three T_4_ data points was divided by the average of all three T_0_ data points for that treatment. The data from the RPL24 shRNA-treated cells was then normalized to that from the control cells. Data is represented by the mean and standard deviation of triplicates.

### Cell lysis and western blotting

Cells were lysed in RIPA buffer (10 mM Tris-HCL (pH 8.0), 1mM EDTA, 0.5 mM EGTA, 1% triton X-100, 0.1% sodium deoxycholate, 0.1% SDS, 140 mM NaCl, 20 mM NaF, Complete EDTA-free protease inhibitor tablets (Roche Diagnostics Corp., Basel, Switzerland) and the phosphatase inhibitor cocktail PhosSTOP (Roche)), the latter two as indicated by the manufacturer's protocol. Equal amounts of protein were diluted in 2X sample buffer. Immunoblots on PVDF (Polyvinyldene Fluoride) membranes were blocked with nonfat milk in tris-buffered saline with 0.05% tween-20 (TBST). The following antibodies were incubated with membranes in 5% nonfat milk in TBST: RPL24 (Proteintech, Chicago, IL), Cyclin D1, Rack1, RPL4 (Santa Cruz Biotechnology, Santa Cruz, CA), NBS1, GAPDH (EMD Millipore Corporation, Chicago, IL), Survivin, β-tubulin, acetyl-lysine, eIF6, acetyl-H2B, H2B, (Cell Signaling Technology, Boston, MA), acetyl-tubulin, tubulin (Sigma Aldrich (St. Louis, MO)). Densitometry was performed using ImageJ software.

### Isolation of ribosomes

Cells, plated at ~90% confluency, were treated as indicated. After treatment, cells were treated with 50 μg/ml cycloheximide for 15 minutes. Cells were lysed with a buffer containing 10 mM HEPES, 10 mM KCl, 75 mM NaCl, 10 mM MgCl_2_, 0.35% NP40, pH 7.9 supplemented with Complete EDTA-free protease inhibitor tablets, PhosSTOP phosphatase inhibitor tablets (Roche) per manufacturer's instructions, 50 μg/ml cycloheximide, SUPERase RNase inhibitors (Life Technologies) per manufacturer's instructions, 15 μM TSA, and 5 mM nicotinimide to inhibit HDACs. Supernatants were collected as cytoplasmic preparations. Where indicated, pellets containing nuclei were resuspended in RIPA buffer (described above). The suspension was spun at 13,000 rpm for 5 min and supernatants were collected as nuclear preparations.

Ribosomes were subsequently isolated from cytoplasmic preparations as described previously [28]. Briefly, lysates were layered on top of a 12% and 33% discontinuous sucrose gradient and spun at 38,000 rpm for 2 h. The resulting polysome pellet was then resuspended, stripped of RNA with acetic acid, and then pelleted with acetone. The pellet was then resuspended in 8 M urea, 2% CHAPS, and 25 mM dithiothreitol (DTT).

Polysome profiles to separate the 40S, 60S, 80S and polysomes were carried out by layering cell lysates over a continuous 10-50% sucrose gradient and spun at 38,000 rpm for 2 h as previously described [29, 30]. Fractions were collected using a Retriever 500 fraction collector with a UV (UA6) detector (ISCO Teledyne (Lincoln, Nebraska)).

### Visualization of crystallography data

Pymol software (Schrodinger, Mannheim, Germany) was used to visualize RPL24 and eIF6 on previously published crystallography data of the *Tetrahymena thermophilia* 60S ribosomal subunit (human gene names used) bound to eIF6 [13].

### Drugs

TSA was obtained from Sigma Aldrich, Entinostat from Syndax (Walthan, MA) and Tubacin (Caymen Chemicals, Ann Arbor, MI). ACY-775 was obtained from Acetylon Pharmaceuticals (Boston, MA).

### Mass spectrometry

To prepare polysome samples for mass spectrometry, cells were treated with HDACi and polysome pellets were prepared using a discontinuous sucrose gradient as described above. Protein concentration was determined using the Pierce™ BCA Protein Assay Kit (Thermo Scientific) and equal amount of protein were trypsin digested. Acetyl-lysine immunoprecipitations were carried on resultant peptides out using an antibody from Cell Signaling Technology. Acetyl-proteins were then eluted, extracted, and desalted.

To determine the acetylation status of 60S subunit proteins, cells were treated with HDACi and polysome profiles were performed as described above. The four fractions representing the 60S subunit were identified, via western blots, for the absence of Rack1 (40S marker) and the presence of RPL24 (60S marker). Those 60S fractions were then TSA precipitated and reconstituted in 2% SDS. 60S samples (normalized by protein loading) were resolved using 4-12% Bis-Tris gels and stained with Imperial Protein Stain (Thermo). Gel bands were excised, diced into small pieces, destained, reduced with 10 mM dithiothreitol, and alkylated with 5 mM iodoacetamide. In-gel trypsin digestion was performed using a 1:20 enzyme to protein ratio for 16 h at 37^o^C. Resultant peptides were extracted and desalted.

Three biological replicates of polysome or 60S samples were then analyzed by LC-MS/MS using a quadrupole time-of-flight (QqTOF)TripleTOF 5600 mass spectrometer (AB SCIEX, Dublin, CA) coupled to an Eksigent (Dublin, CA) nanoLC Ultra, 2D plus. Briefly, the resulting peptides were chromatographically separated on a C-18 reversed-phase analytical column (75 μm I.D.) connected to the TripleTOF 5600 operating in data dependent mode (1 MS1 survey scan followed by 30 MS/MS scans per 1.8 second acquisition cycle) [31]. Mascot v2.3.02 and ProteinPilot v4.5 data base searches were employed for peptide identification (Table S1, Table S2) using a false discovery rate analysis (FDR) of 0.01. For MS/MS spectral data of acetylated peptides see Figures S3 and S4. Moreover, more detailed interactive viewing of spectral libraries is available at the Panorama webserver (University of Washington, Seattle), at ‘https://daily.panoramaweb.org/labkey/project/Gibson/Polysomes_Benz2/begin.view?’.

Quantitative data analysis of acetyl-lysine peptides was performed by integration of selected molecular ion intensities using Skyline MS1 Filtering as previously described [31]. The average signal intensity, as determined by the area under the curve (AUC) of the LC chromatogram, of the replicate biological samples was calculated. The amount of acetylated peptide normalized to total protein loaded onto the gel for each condition was determined and the fold induction upon TSA treatment was then calculated.

## CONCLUSIONS

This is the first study implicating a role for RPL24 expression in human breast tumors and in regulating protein translation necessary for breast tumorigenesis. We demonstrate that RPL24 depletion decreases the level of proteins synthesized by cap-dependent translation essential for cancer cell growth, survival and genome repair, without altering the synthesis of housekeeping proteins. Polysome profiling indicates that this partial translation defect might be related to the control of eIF6 release from the 60S subunit, inhibiting assembly of 80S ribosomes and polysomes. Furthermore, we show that HDACi and more specifically, inhibition of HDAC6, rapidly induces acetylation of multiple ribosomal proteins, and that RPL24 acetylation at K27 is associated with impairment in ribosome assembly similarly induced by RPL24 depletion. Therefore, our findings point toward the existence of an endogenous, HDAC6- and protein deacetylation-dependent polysome assembly mechanism that can regulate oncogenic growth and potentially serve as a novel cancer therapeutic target.

### Competing Interests

The authors declare no conflicts of interest.

### Author's Contributions

Conceived and designed the experiments: KAW, AK, GKS, JMH, BWG, CCB. Performed experiments: KAW, AK, GKS, DER, BSG, MAY, IMH. Analyzed and interpreted data: KAW, AK, GKS, CY, BS, JMH, BWG, CCB. Wrote the paper: KAW, AK, BS, CCB.

## SUPPLEMENTARY METHODS, MATERIAL, FIGURES AND TABLES






