# Supplementary material for: RPL24: a potential therapeutic target whose depletion or acetylation inhibits polysome assembly and cancer cell growth
Source: Oncotarget. 2014 Jun 12;5(13):5165–76. doi: 10.18632/oncotarget.2099 (PMC4148130; doi:10.18632/oncotarget.2099)
Supplement: Supplementary file 1 [file oncotarget-05-5165-s001.pdf]

RPL24: a potential therapeutic target whose depletion or acetylation inhibits polysome assembly and cancer cell growth

Supplemental Materials and Methods

Cloning

Full length (amino acids 1-154) and truncated (amino acids 1-137) RPL24 was PCR amplified from pCMV6-XL5-RPL24 (OriGene, Rockville, MD) using primers containing EcoR1 and Not1 sites. The amplicons were then cloned into the pCMV6-KanNeo vector (OriGene) using standard cloning techniques.

Transfection

293T cells (ATCC) were transfected with Lipofecatmine 2000 (Life Technologies) according to the manufacturer's protocols.

RNA isolation and northern blots

Cells were harvested and RNA was extracted using Trizol (Life Technologies) per manufacturer's protocol. Northern blots were performed as previously described [25, 36]. Briefly, RNA was then electrophoresed into 1% agarose-formaldehyde gels and transferred onto PVDF membranes. Membranes were then hybridized with ^{32}P-labelled cDNA probes for HER2 or GAPDH, washed, and visualized by autoradiography.

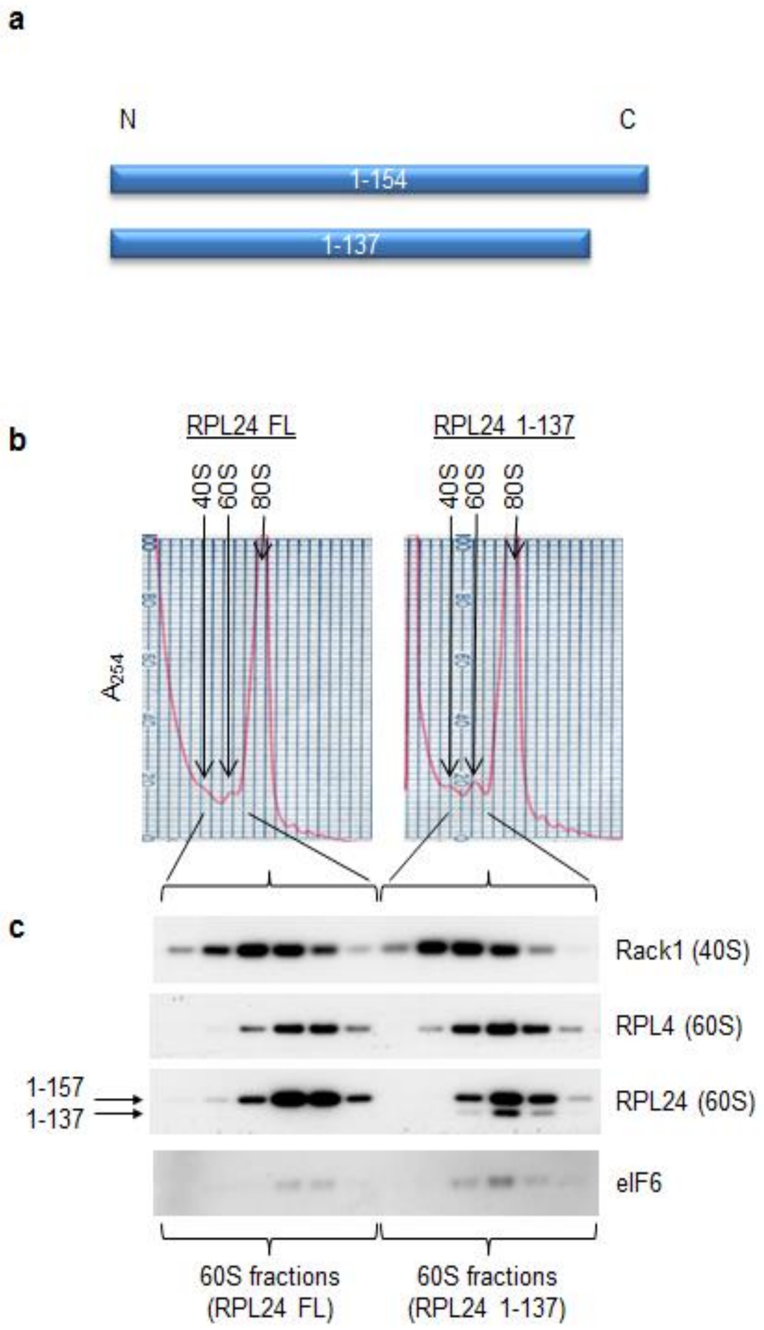

Figure S1: Expression of truncated RPL24 increases association of eIF6 with 60S fractions in 293T cells. (A-C) 293T cells were transfected with either full length (amino acids 1-154) or truncated RPL24 (amino acids 1-137). (B) Two days later, cells were lysed and polysome profiles were performed. (C) W estern blots using antibodies toward the indicated proteins were performed on 60S fractions.

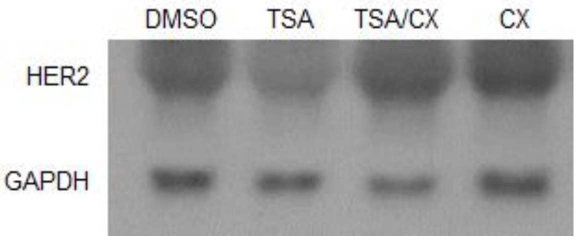

Figure S2: TSA-induced HER2 mRNA decay is abrogated by cycloheximide treatment. SKBR3 cells were treated with TSA (1 µM, 6 h) and/or cycloheximide (CX, 50 µg/ml, 6 h) or with the respective vehicle controls. RNA was isolated and northern blotted for HER2 and GAPDH transcript levels as shown.

a

MS/MS spectra of HMYHSLYL**Kac**VK K-126 P84098 RL19_HUMAN

m/z 487.60 +++

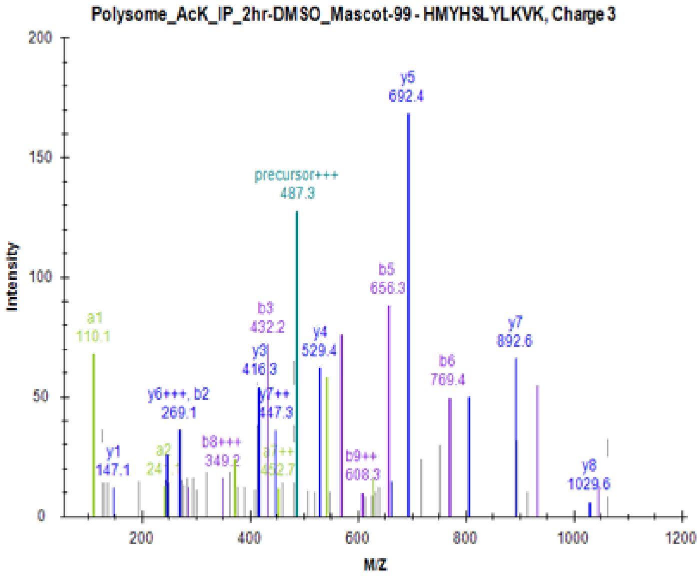

b

MS/MS spectra of ILMEHIH**Kac**LK K-144 P84098 RL19_HUMAN

m/z 435.26 +++

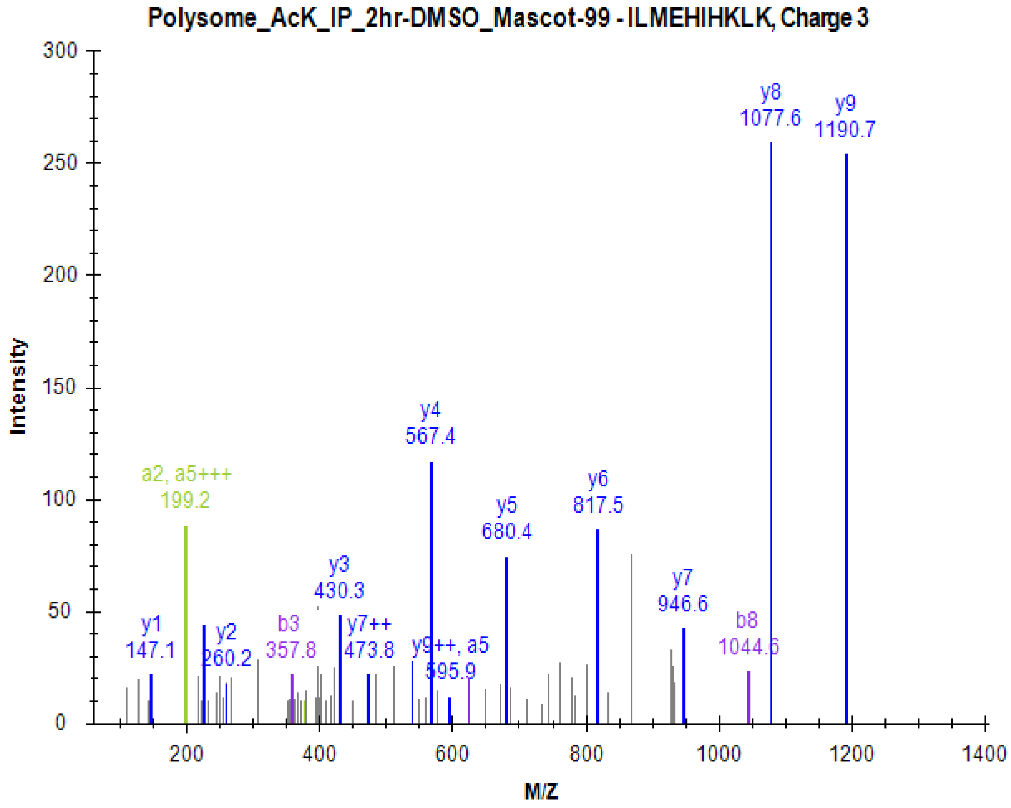

c

MS/MS spectra of ITVTSEVPFS**Kac**R K-80 P35268 RL22_HUMAN

m/z 469.27 +++

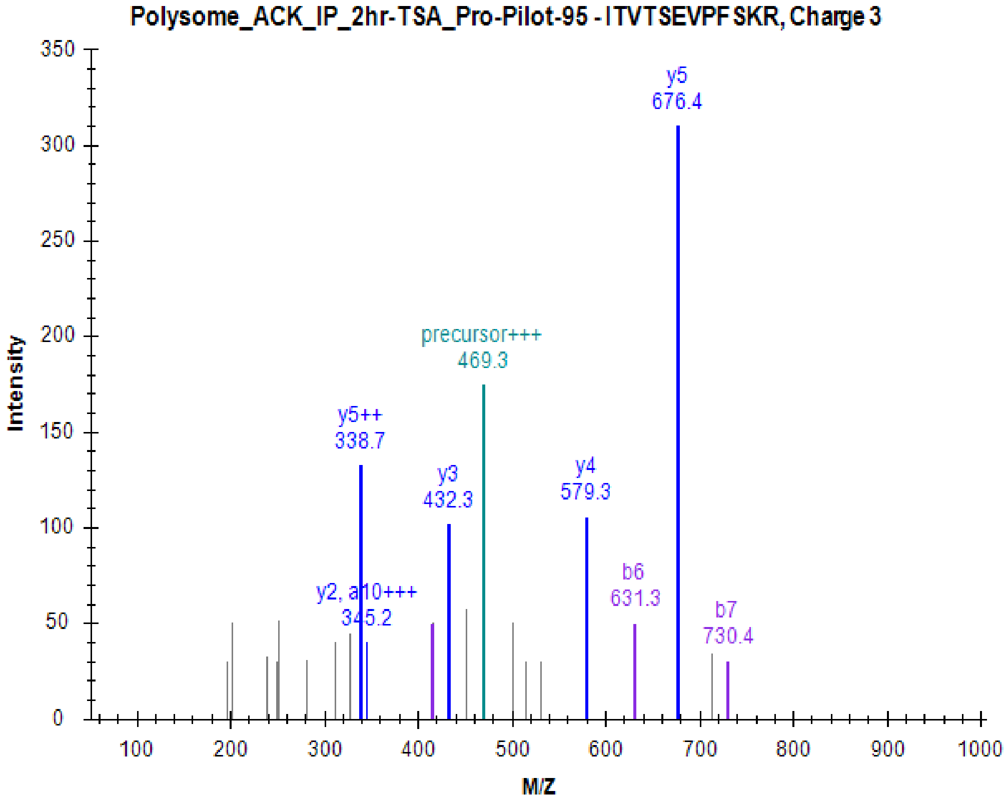

d

MS/MS spectra of AITGASLADIMA**Kac**R K-93 P83731 RL24_HUMAN

m/z 730.40++

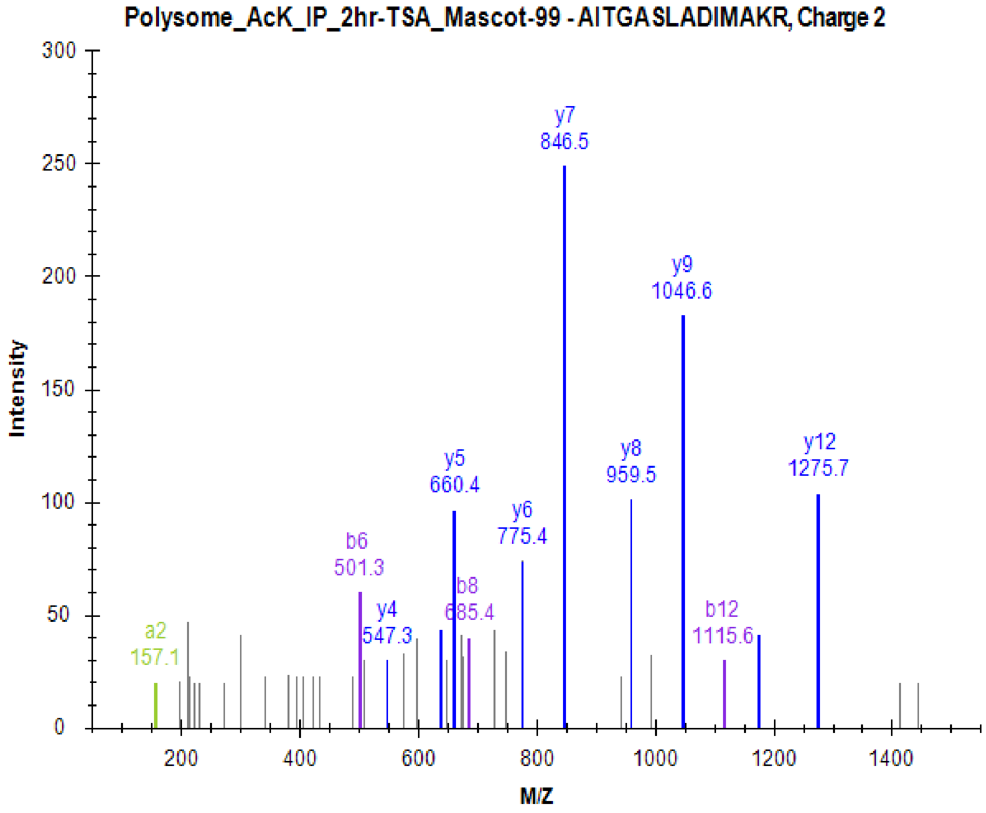

e

MS/MS spectra of DVFRDPAL**Kac**R K-107 P61353 RL27_HUMAN

m/z 420.23 +++

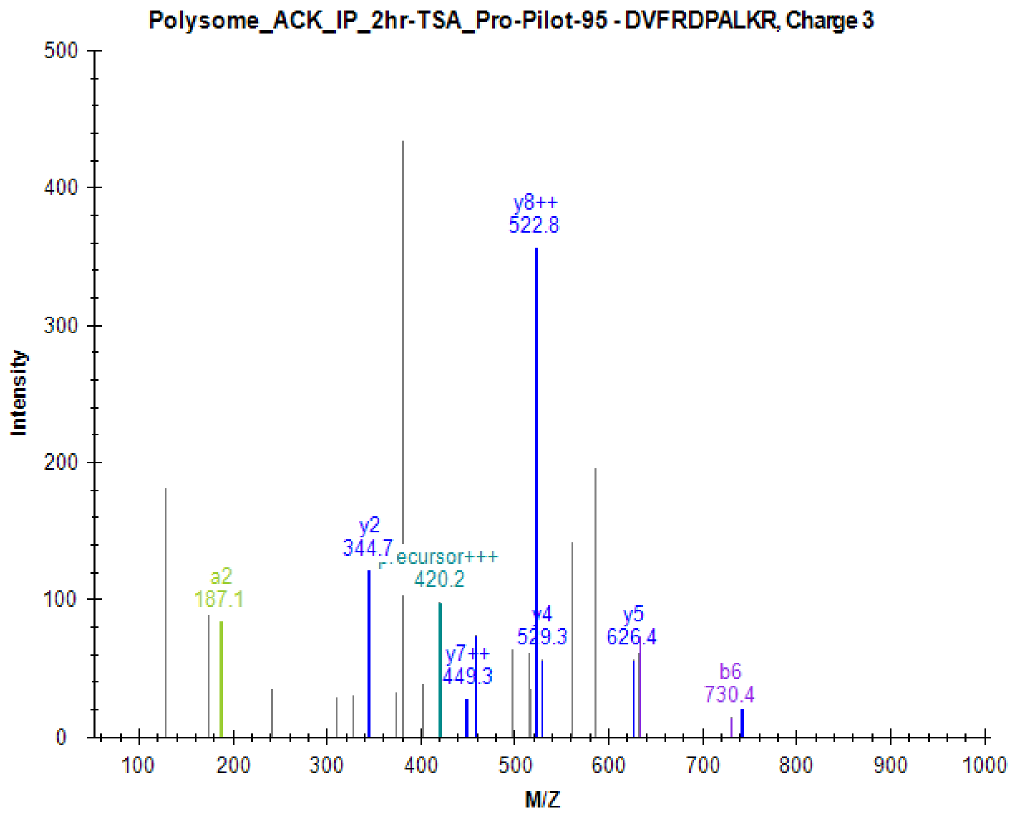

f

MS/MS spectra of GVVVVI**Kac**R K-65 P46779 RL28_HUMAN

m/z 456.31 ++

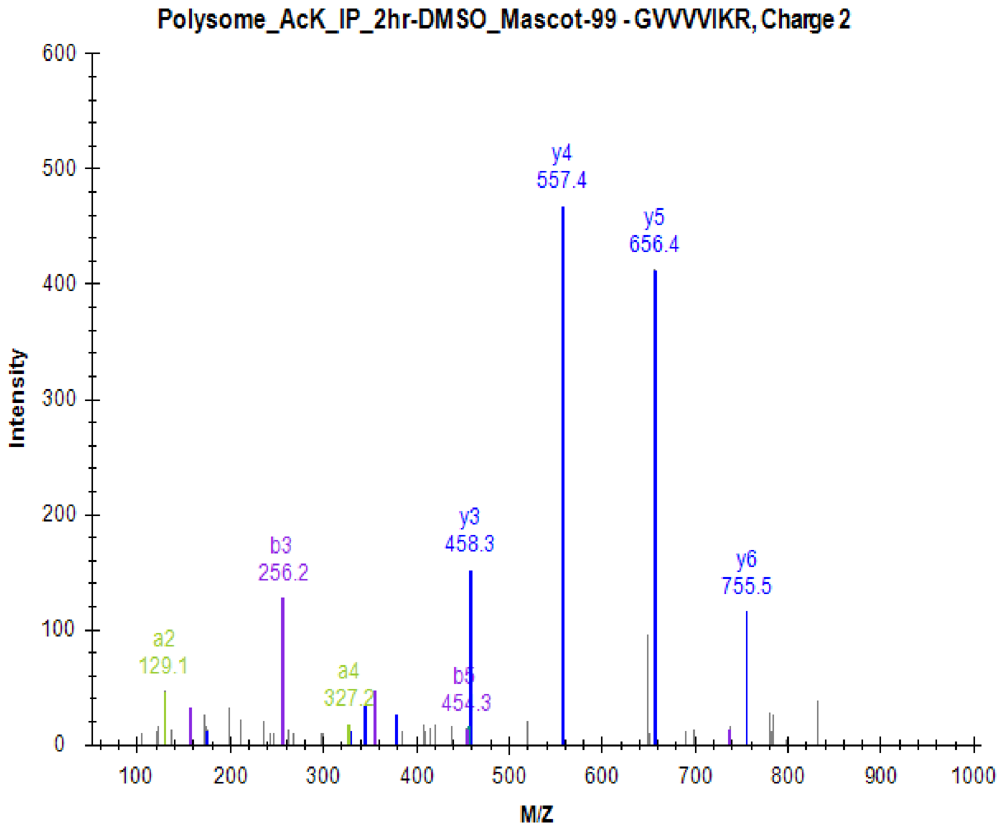

g

MS/MS spectra of TVFAEHISDEC**Kac**R K-115 P39023 RL3_HUMAN

m/z 545.26 +++

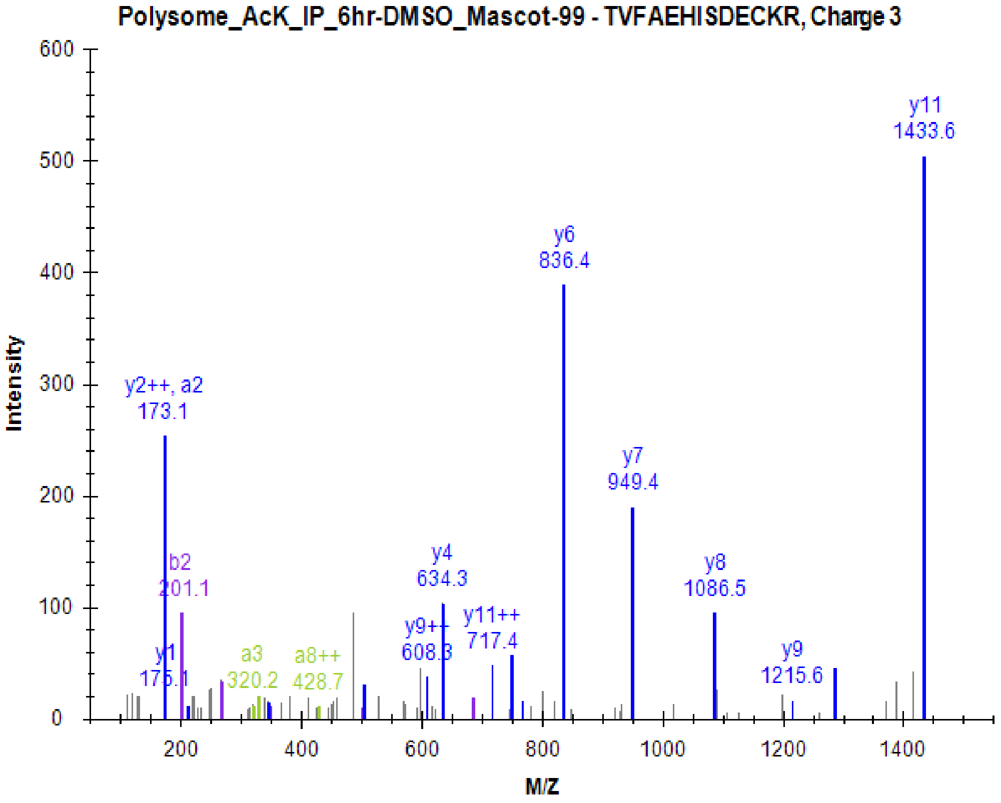

h

MS/MS spectra of DETEFYLG**Kac**R K-45 P18077 RL35A_HUMAN

m/z 650.31 ++

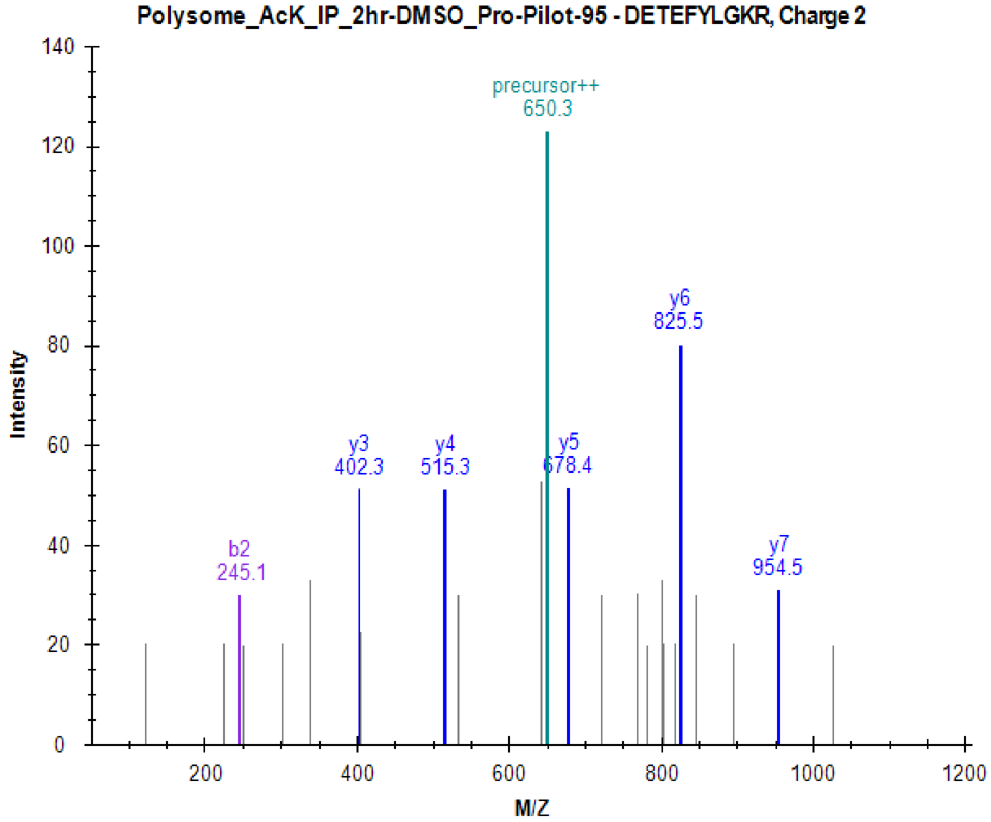

i

MS/MS spectra of VGIVG**Kac**YGTR K-13 P61513 RL37A_HUMAN

m/z 546.31 ++

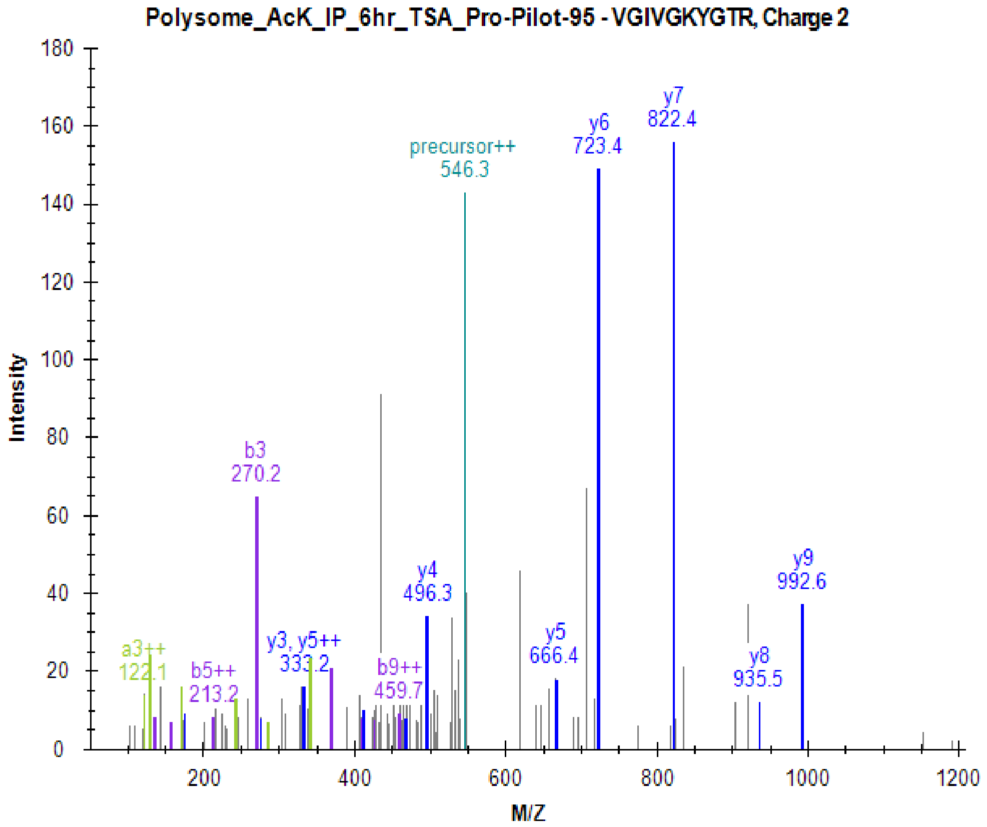

j

MS/MS spectra of FIDTTS**Kac**FGHGR K-373 Q92901 RL3L_HUMAN

m/z 469.91 +++

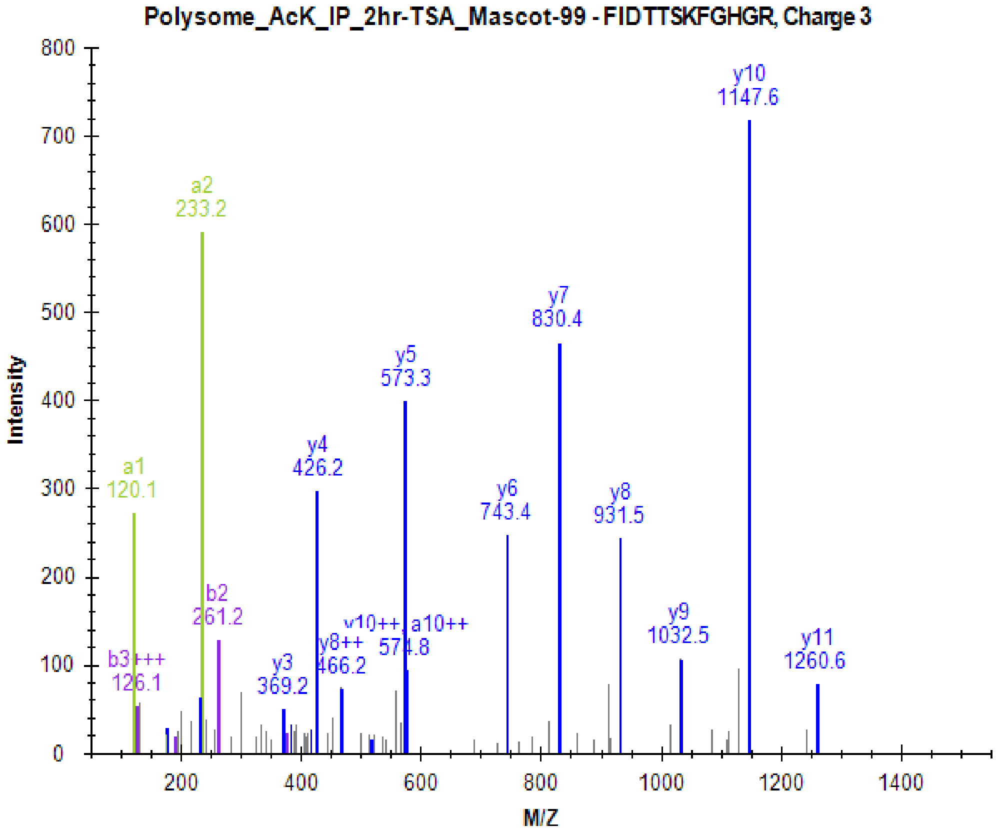

k

MS/MS spectra of THQ**Kac**FVIATSTK K-192 Q02878 RL6_HUMAN

m/z 468.26 +++

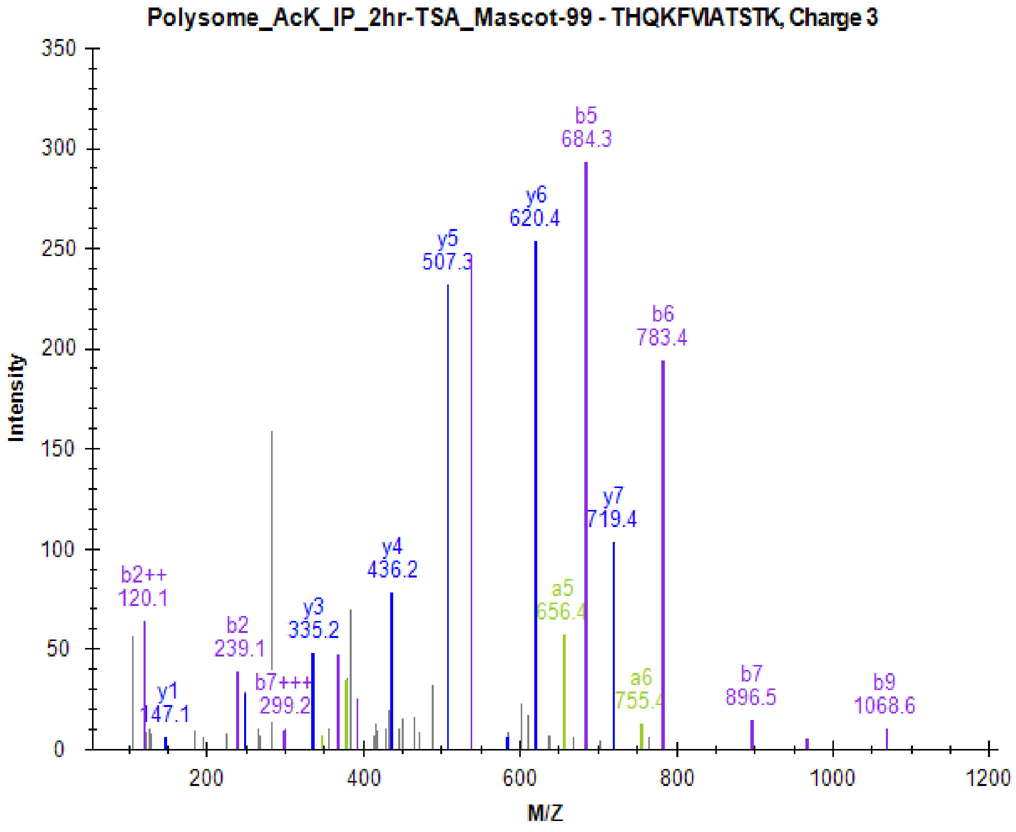

I

MS/MS spectra of NFGIGQDIQP**Kac**R K-48 P62ac4 RL7A_HUMAN

m/z 472.25 +++

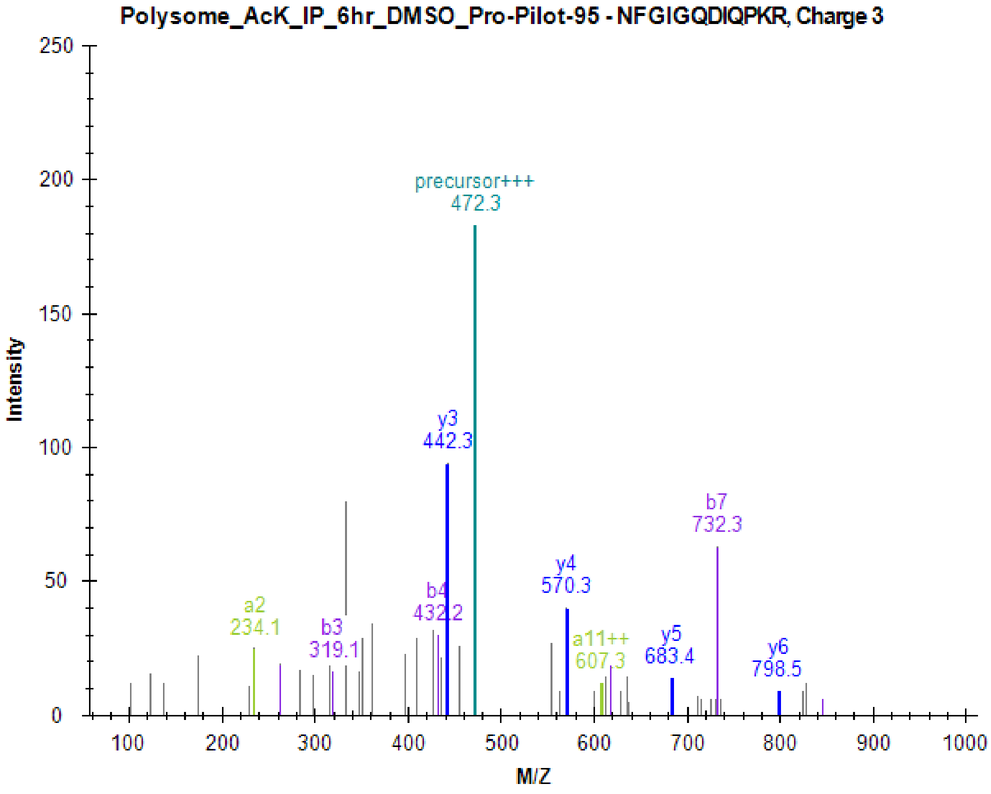

m

MS/MS spectra of AGVNTVTTLVEN**Kac**K K-150 P62ac4 RL7A_HUMAN

m/z 505.95 +++

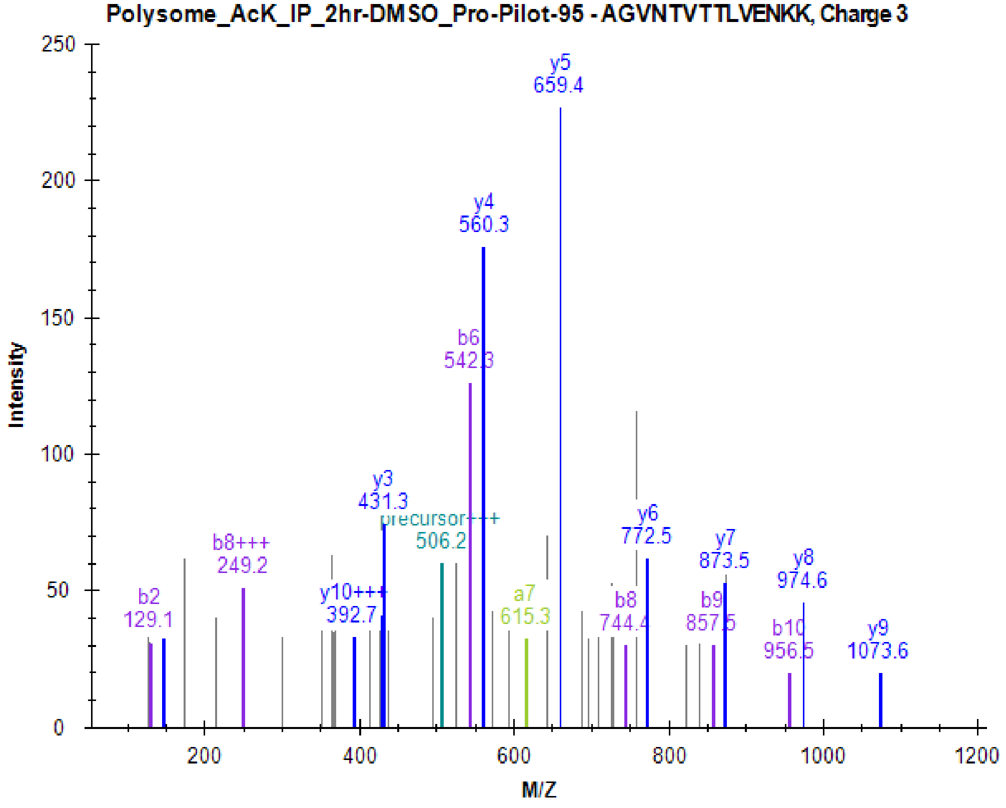

n

MS/MS spectra of GLAPDLPEDLYHLI**Kac**K K-93 P62277 RS13_HUMAN

m/z 622.01 +++

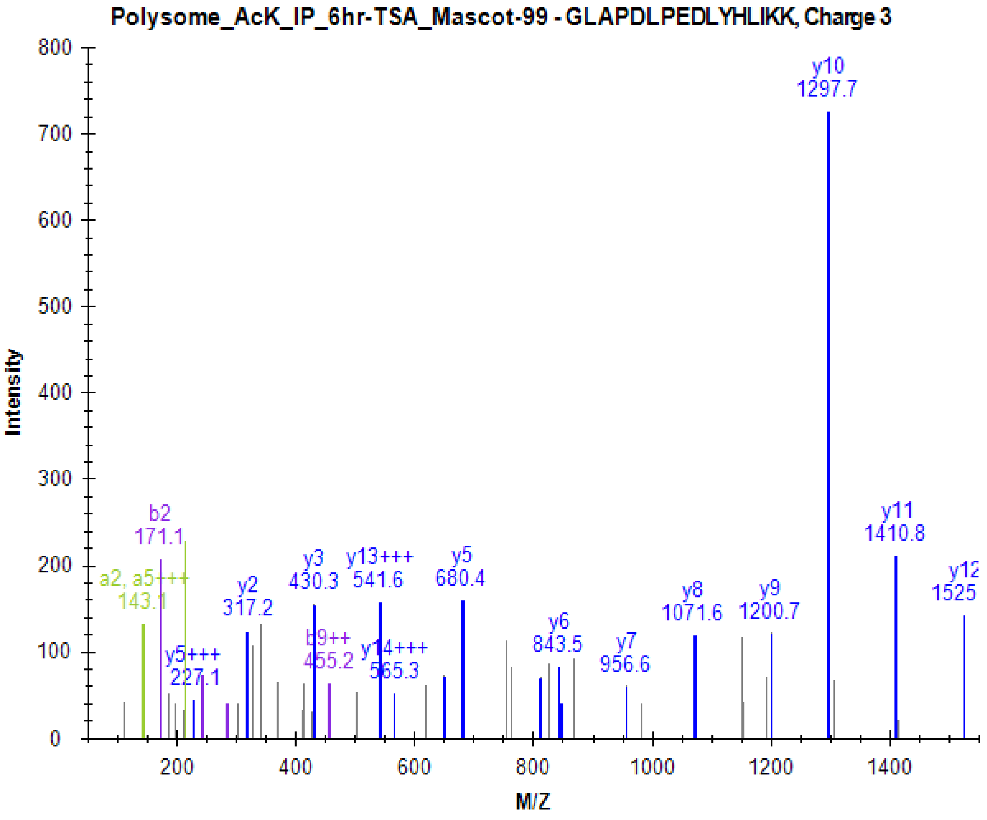

o

MS/MS spectra of IAGYVTHLM**Kac**R K-59 P0CW22 RS17L_HUMAN

m/z 444.25 +++

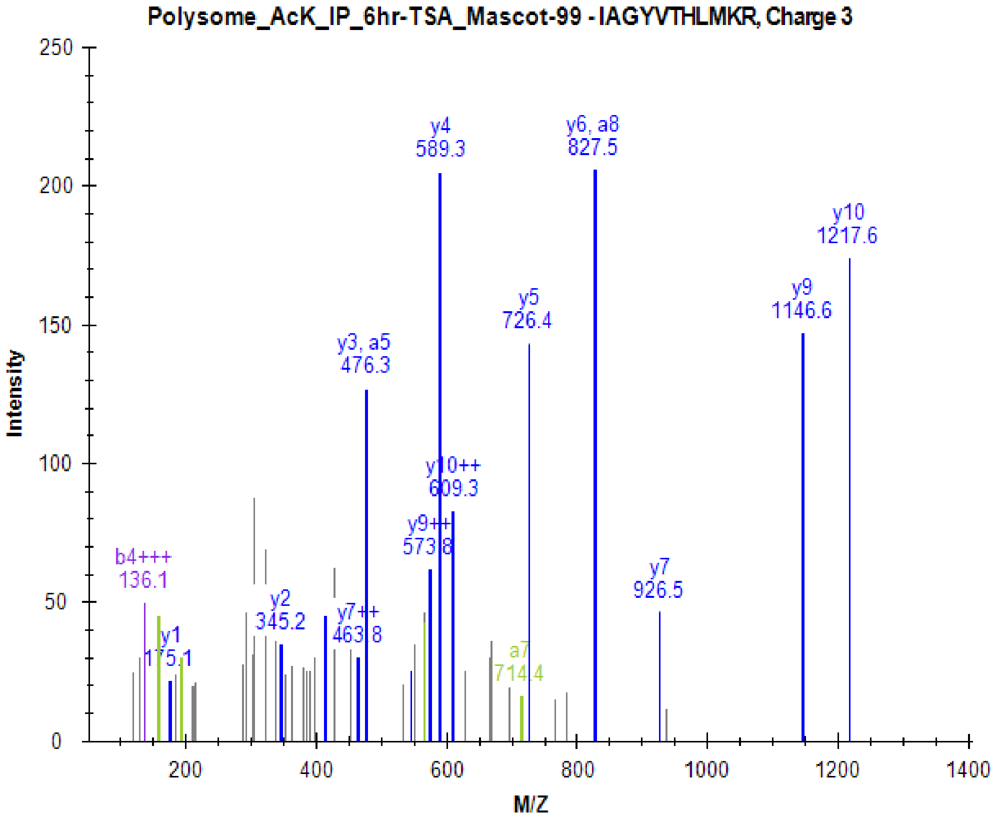

p

MS/MS spectra of VANVSLLALY**Kac**GK K-135 P62266 RS23_HUMAN

m/z 709.43 ++

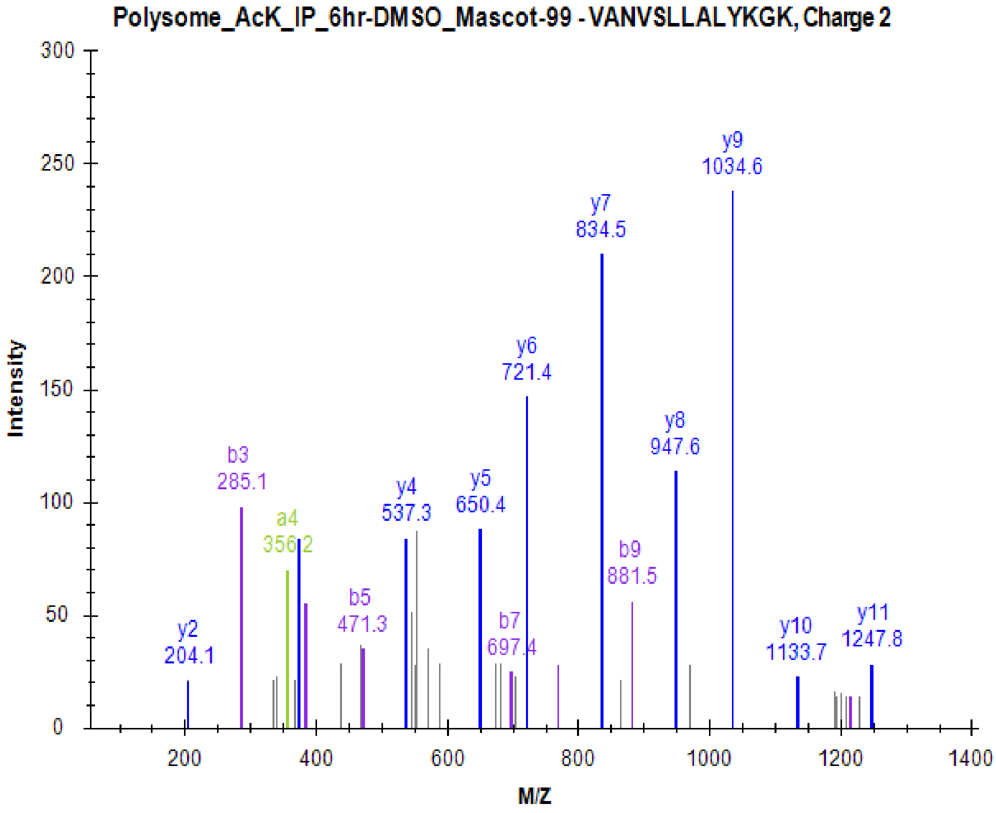

q

MS/MS spectra of LAVL**Kac**YYK K-104 P62979 RS27A_HUMAN

m/z 520.31 ++

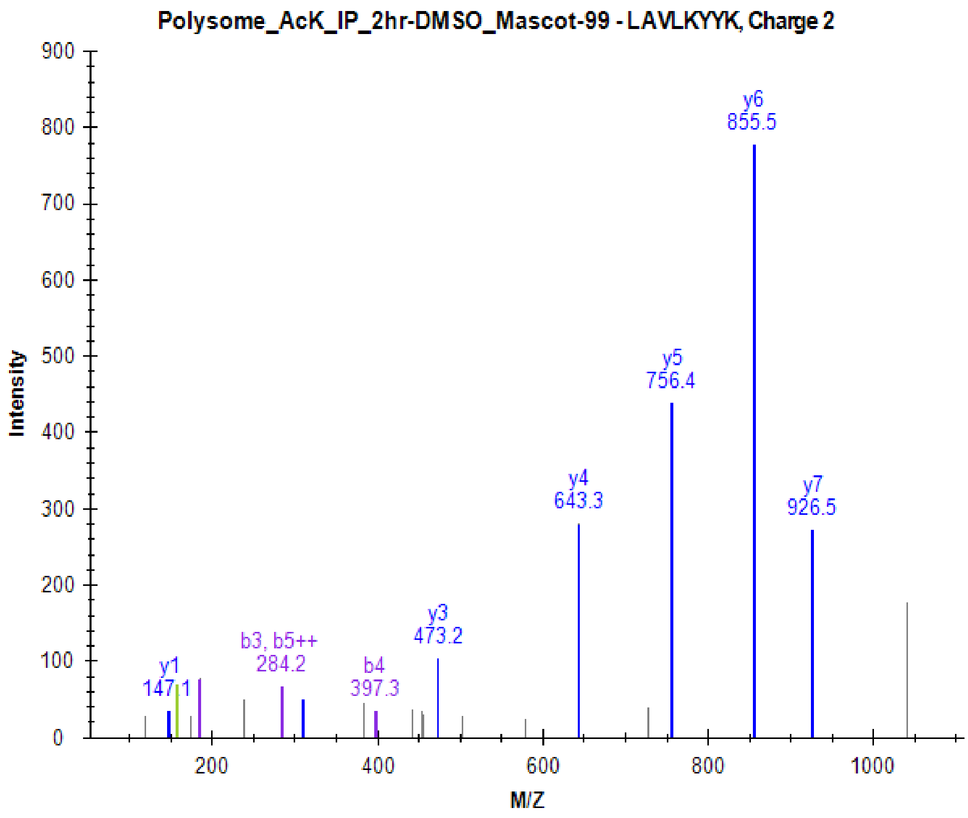

Figure S3a-q: ESI-MS/MS spectra for lysine acetylated peptides obtained from polysome preparations. For each acetylated peptide the annotated ESI-MS/MS spectrum is displayed, the peptide sequence is indicated including the acetylated lysine residue 'Kac' within the sequence, and the lysine acetylation site (K residue number) is provided. In addition, SwissProt accession numbers and the corresponding protein names are listed. The precursor ion *m/z* value that was selected for MS/MS, as well as the charge state, is displayed above the spectrum. Fragment ions are annotated as y or b ions within the spectrum above the observed fragment ion *m/z* values. All spectra were acquired on a quadrupole time-of-flight (QqTOF) TripleTOF 5600 mass spectrometer.

b

MS/MS spectra of AITGASLADIMA**Kac**R Kac-93 P83731 RL24_HUMAN

m/z 730.40 ++

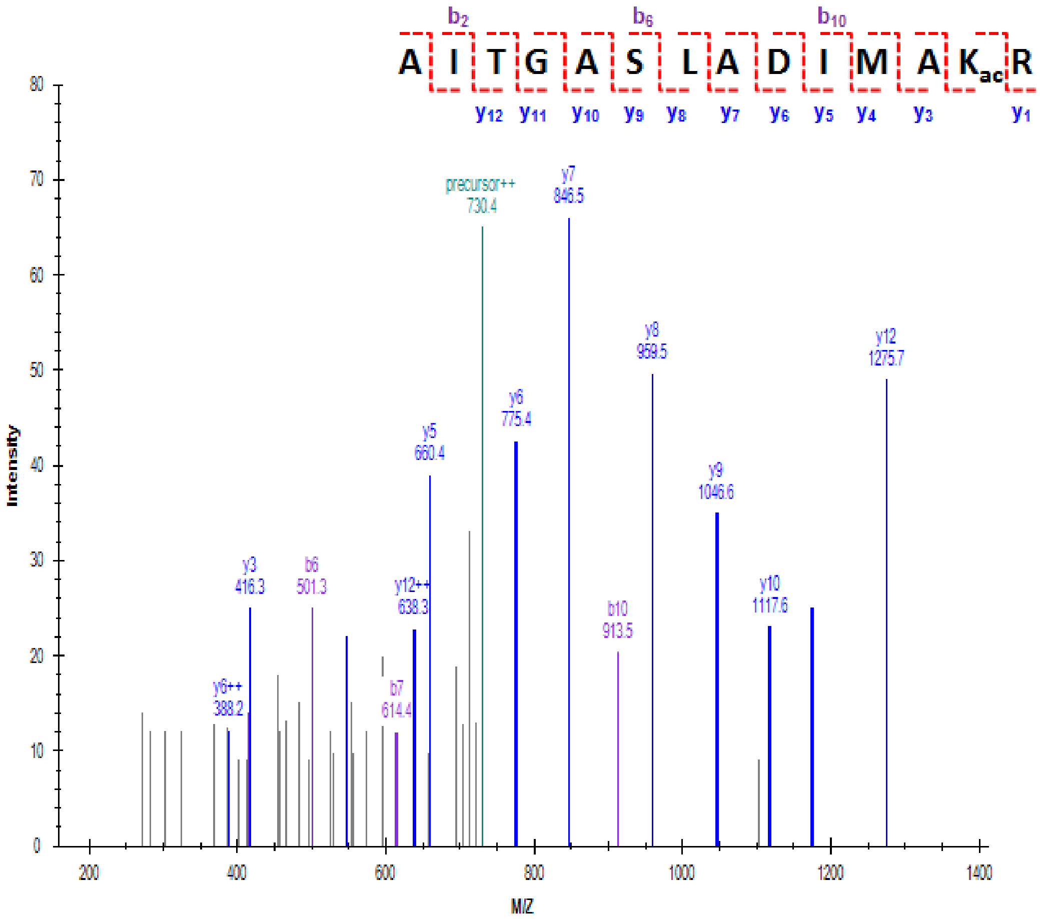

Figure S4: ESI-MS/MS spectra for lysine acetylated peptides obtained from 60S preparations. A representative MS/MS spectra for the acetyl-K27 (a) and acetyl-K93 (b) sites of 60S-associated RPL24. Peptide $[M + 2H]^{2+}$ precursor ions *m/z* 705.37 and *m/z* 730.40 were fragmented by collision-induced dissociation (CID). The y-type and b-type ions were used to identify the peptide sequence and locate the acetylation site.
